# Improved *in vitro* Efficacy of Baloxavir Marboxil Against Influenza A Virus Infection by Combination Treatment With the MEK Inhibitor ATR-002

**DOI:** 10.3389/fmicb.2021.611958

**Published:** 2021-02-12

**Authors:** Hazem Hamza, Mahmoud M. Shehata, Ahmed Mostafa, Stephan Pleschka, Oliver Planz

**Affiliations:** ^1^Department of Immunology, Institute for Cell Biology, Eberhard Karls University of Tübingen, Tübingen, Germany; ^2^Virology Laboratory, Environmental Research Division, National Research Centre, Giza, Egypt; ^3^Center of Scientific Excellence for Influenza Viruses, National Research Centre, Giza, Egypt; ^4^Institute of Medical Virology, Justus Liebig University Giessen, Giessen, Germany; ^5^German Center for Infection Research (DZIF), Partner Site Giessen, Giessen, Germany

**Keywords:** influenza virus, antivirals, combination treatment, MEK inhibitors, antiviral resistance, Baloxavir Marboxil

## Abstract

Currently, all available antiviral drugs against influenza virus (IV) that target the virus proteins directly, like Baloxavir acid (BXA), lead to viral resistance. Therefore, cellular mechanisms and factors essential for IV replication are promising antiviral targets. As IV strongly depends on the virus-induced Raf/MEK/ERK signal pathway for efficient generation of infectious progeny virions, this pathway represents an important target. We aimed to determine whether the MEK inhibitor ATR-002 (PD0184264) is able to impair replication of BXA-resistant influenza A virus (IAV) and whether a treatment combining BXA and ATR-002 improves the therapeutic efficiency *in vitro*. A549 cells infected with different IAV strains including BXA-resistant variants were treated with ATR-002 or BXA and the effect on virus titer reduction was determined. The synergistic effect of ATR-002 and BXA was also analyzed using different evaluation methods. The data demonstrated that ATR-002 has a significant and dose-dependent inhibitory effect on IAV replication across different strains and subtypes. IAV with the PA-I38T mutation shows resistance against BXA, but is still susceptible toward ATR-002. The combination of ATR-002 and BXA exhibited a synergistic potency reflected by low combination index values. In conclusion, we show that ATR-002 permits to counteract the limitations of BXA against BXA-resistant IAV. Moreover, the results support the use of ATR-002 (i) in a mono-therapy, as well as (ii) in a combined approach together with BXA. These findings might also apply to the treatment of infections with IAV, resistant against other direct-acting antiviral compounds.

## Introduction

Influenza virus (IV) drug resistance represents a significant health threat. The hetero-trimeric RNA-dependent RNA polymerase (RdRp) complex of IV is comprised of three subunits: PB2, PB1, and PA, each with specific functions (Obayashi et al., 2008; Muhlbauer et al., 2015). The PA subunit contains the endonuclease that cleaves the CAP-structure (cap-snatching) from cellular mRNAs, which is then used by the RdRp to generate capped primers for viral mRNA synthesis (Fodor, 2013; Pleschka, 2013; Pflug et al., 2017). Together with the viral RNA (vRNA) and the nucleocapsid protein (NP), the RdRp forms the biological active viral ribonucleoprotein complex (vRNP), which transcribes and replicates the viral genome within the nucleus of the infected cell. The virus can escape selective pressures as the RdRp lacks a proof-reading function. Accumulating point mutations constantly alter the genome sequence, allowing the virus to generate a multitude of genetic and antigenic variants. Furthermore, the segmented genome permits the exchange of vRNA segments between different viruses upon co-infection, leading to new viruses with altered characteristics (Lauring and Andino, 2010).

Currently, three classes of FDA-approved antiviral drugs are worldwide available, M2 channel ion blockers (Adamantanes), neuraminidase inhibitors (NAIs), and polymerase inhibitors. Due to the widespread resistance of circulating viruses, adamantanes are not recommended presently for clinical use and NAIs are the only effective antivirals used in most countries. However, the global circulation of NAIs-resistant IV variants remains a threat (Duwe, 2017; Hayden and Shindo, 2019). Several new antiviral drugs that target RdRp functions are in clinical development; these include Pimodivir^®^, a PB2 inhibitor (Clark et al., 2014), and Favipiravir^®^, a PB1 inhibitor (Vanderlinden et al., 2016). Recently, the cap-dependent endonuclease inhibitor Baloxavir acid (BXA) has been licensed in Japan and the United States for the treatment of uncomplicated influenza and high-risk patients (Hayden et al., 2018; Noshi et al., 2018; Hirotsu et al., 2019). Unfortunately, already during clinical trials, BXA-resistant variants were detected in the post-treatment monitoring. The PA-I38T substitution strongly reduced PA susceptibility to BXA (Jones et al., 2018; Omoto et al., 2018; Hirotsu et al., 2019). Even though these mutants showed impaired replicative fitness, other studies have well demonstrated that endonuclease-resistant variants with only modest effects on viral fitness can emerge *in vitro* and *in vivo* (Song et al., 2016).

However, all IVs also depend on essential host factors including cellular signaling cascades. Importantly, these are not virus-encoded and are therefore not prone to mutations caused by the RdRp. Therefore, such essential host factors are promising antiviral targets. Several studies demonstrate that blocking specific signaling pathways not only reduced viral titers but also led to immunomodulation regulating an uncontrolled host response without the occurrence of viral resistance (Ludwig, 2011; Pinto et al., 2011; Lee and Yen, 2012; Planz, 2013). The mitogen-activated protein kinase (MAPK) cascade is closely associated with cell proliferation and its inhibition is a cornerstone in different cancer therapies (Sebolt-Leopold, 2004; Lorusso et al., 2005; Fremin and Meloche, 2010). Upon infection, the virus induces the activation of the Raf/MEK/ERK signaling pathway, which is pivotal for efficient IAV replication leading to enhanced RdRp activity (Marjuki et al., 2007) and temporal/spatial coordination of the nuclear vRNP export essential for efficient production of infectious progeny virions (Marjuki et al., 2006). Therefore, blockade of this pathway by specific MEK-inhibitors leads to nuclear vRNP retention (Pleschka et al., 2001; Droebner et al., 2011) and different MEK-inhibitors, which are already approved for cancer treatment (Trametinib®) or used earlier in clinical trials (CI-1040), have shown a high anti-IV activity (Haasbach et al., 2017; Schrader et al., 2018). However, despite the antiviral activity of CI-1040, phase I and pharmacodynamics studies reported that the CI-1040 plasma concentrations increased in less than a proportional manner and appeared to plateau even when administrated at high doses. Yet, it was observed that the active metabolite ATR-002 (PD0184264) was present in the plasma at higher concentrations than the parent compound (Lorusso et al., 2005). However, the pharmaceutical development of ATR-002 was not further promoted as an antitumor agent (Sebolt-Leopold et al., 1999; Tecle et al., 2009). Nevertheless, we previously explored the antiviral potential of ATR-002 compared to CI-1040 and could demonstrate the *in vivo* superiority of ATR-002 over CI-1040 as an antiviral compound, despite its weaker membrane permeability (Laure et al., 2020).

In addition to mono-therapies, combinatorial therapies with drugs that either have similar or different activities, targeting various viral proteins or host factors, might further reduce the emergence of drug-resistant strains (Hayden, 2009; Govorkova and Webster, 2010). Therefore, not only the simultaneous application of two drugs, both *in vivo* and *in vitro* (Haasbach et al., 2013; Belardo et al., 2015; Pires de Mello et al., 2018), but also triple combinations have been investigated (Nguyen et al., 2010; Kim et al., 2011).

Giving the frequency of viral resistance to BXA, we here evaluated the anti-IAV potential of the MEK inhibitor ATR-002 compared to BXA against wild type and BXA-resistant strains. In terms of improved therapeutic efficacy, treatment applying ATR-002 together with BXA was also conducted to investigate whether the combined agents would reduce the replication of different influenza A viruses (IAV) synergistically, additively or, antagonistically.

## Materials and Methods

### Drugs

ATR-002 (PD0184264) [2-(2-chloro-4-iodophenylamino)-*N*-3,4-difluoro benzoic acid], the active metabolite of CI-1040, was synthesized at ChemCon GmbH (Freiburg, Germany). Baloxavir acid (BXA) was purchased from Hycultec GmbH (Cat: HY-109025) and prepared at a stock concentration of 1 mM according to the manufacturer’s instructions.

### Cells and Viruses

Human lung adenocarcinoma cells (A549, ATCC^®^ CCL185^TM^) and Madin-Darby canine kidney cells (MDCK II, ATCC^®^ CRL2936^TM^) were cultured in IMDM medium. Human embryonic kidney cells expressing the SV40 large T-antigen (HEK 293T, ATCC^®^ CRL-3216^TM^) were maintained in DMEM Medium. Following wild type influenza A virus strains were used: A/Mississippi/3/2001 (H1N1), A/Perth/265/2009 (H1N1pdm09), A/Puerto Rico/8/34 (H1N1), and A/Victoria/03/75 (H3N2).

### Generation of Recombinant Viruses

Two recombinant wild type influenza virus rgA/Giessen/6/2009 (H1N1-WT) and rgA/Victoria/3/75 (H3N2-WT) and their variants rgA/Giessen/6/2009 (H1N1-PA-I38T), rgA/Victoria/3/75 (H3N2-PA-I38T) harboring the BXA-resistance mutation PA-I38T were generated using a reverse genetic system as previously described by Mostafa et al. (2013). Briefly, a complete set of pMP plasmids encoding the eight viral segments of A/Giessen/6/2009 (H1N1) and A/Victoria/3/75 (H3N2) were constructed. Relevant primer pairs (Supplementary Table 1) were used for site-directed mutagenesis to introduce PA segment I38T mutation into the pMP-PA-Gi/-Vic plasmid. PA I38T mutation in pMP-PA-Gi /-Vic and absence of PCR introduced errors in purified plasmid DNA was confirmed by sequencing using gene-specific primers and vector-specific primers (Supplementary Table 2).

### Rescue of Recombinant Viruses

Rescue was performed as previously described (Mostafa et al., 2013). Briefly, the complete set of eight vectors encoding the vRNA of wild type and variant rgA/Victoria/3/75 (rgH3N2-WT, rgH3N2-PA-I38T) and rgA/Giessen/6/2009 (rgH1N1-WT, rgH1N1-PA-I38T) were co-transfected into a co-culture of 293T/MDCK-II cells. At 72 h post transfection, a 500 μl aliquot of each supernatant was used to inoculate MDCK-II cells, which were then incubated for 72 h in the presence of 2 μg/ml TPCK-treated trypsin. Rescued viruses from these cells were stored at −80°C.

After viral RNA extraction, RT-PCR targeting the full PA segment was performed for each recombinant virus. The amplified PA segments were purified and subject to sequencing using specific primers (Supplementary Table 3).

### *In vitro* Replication Kinetics of Recombinant Viruses

Confluent MDCK-II cells monolayers were infected in triplicate with each of the recombinant viruses (MOI: 0.001). After 1 h incubation at 37°C, the inocula were removed, cell monolayers washed with PBS++, and overlaid with infection media (1X DMEM supplemented with P/S, 0.3% BSA, and 2 μg/ml TPCK-treated trypsin). Aliquots, 200 μl each, of supernatant were collected at 6, 24, and 48 hours post infection (hpi). The virus titer was subsequently determined via focus assay (Matrosovich et al., 2006; Ma et al., 2010).

### Virus Yield Reduction (VYR) Assay

IAV susceptibility to either ATR-002 or BXA was determined by measuring the titer reduction (FFU) in the presence of the drugs. Different concentrations of each drug were prepared in DMEM infection media. Confluent monolayers of A549 cells in 24-well plates were infected in triplicates with A/Mississippi/3/2001 (H1N1), A/Perth/265/2009 (H1N1pdm09), A/Puerto Rico/8/34 (H1N1), or A/Victoria/03/75 (H3N2) at an MOI of 0.005, 0.005, 0.001, and 0.001, respectively. After 1h incubation, the inocula were removed, the infection media containing both drug combinations were added and subjected to focus assay after 24 hpi.

### Virus Titration (Focus Assay)

Virus titers were determined using focus assay as described earlier (Matrosovich et al., 2006; Ma et al., 2010). Briefly, MDCK II cells were seeded in 96-well plates and incubated overnight. MDCK II plates were washed with PBS and infected in triplicates with virus-containing supernatants in a 10-fold dilution in PBS/BA (PBS++ containing 0.3% BSA, P/S). After 1 hpi, the inocula were removed and the Avicel^®^ overlay was added to each well. At 24 hpi, the cells were fixed and permeabilized with PBS containing 4% paraformaldehyde and 1% triton-X 100. Afterward, cells were immunostained with mouse anti-IAV nucleoprotein monoclonal antibody (Bio-Rad) followed by goat anti-mouse IgG-HRP (Jackson ImmunoResearch Laboratories). After extensive washing, True Blue^TM^ peroxidase substrate (SeraCare, United States) was added to detect foci and the stained plates were scanned and analyzed. The virus titers in focus-forming unit (ffu/ml) were calculated by multiplying the average ffu for each treatment, the dilution factor, and by the inoculum volume normalization factor. The reduction in virus titers was expressed as (%) by dividing the average ffu for each treatment by the average ffu of the infected non-drug-treated control.

### Analysis of Synergy/Antagonism From Combination Studies

Different concentrations (0.4 – 50 μM) of ATR-002 and (0.008 – 1 nM) BXA were prepared by making fivefold serial dilution in infection media. Combinations between both drugs were tested in a 4 × 4 matrix and all values normalized to Mock-infected control (DMSO). A549 cells were infected with A/Puerto Rico/8/34 (MOI: 0.001). After incubation, the inocula were removed, the confluent monolayers were supplemented with DMEM infection medium containing the tested inhibitor combinations and subjected to focus assay after 24 hpi.

To determine the possible additive and synergistic effects when using combinations of ATR-002 with BXA, the data from VYR assay were analyzed according to the Chou–Talalay model (Chou and Talalay, 1984; Chou, 2010) using CompuSyn software (Chou and Martin, 2005). The software calculates the combination index (CI) for each drug combination, where a CI value < 1 indicates synergy, CI = 1 is additive and CI > 1 indicates antagonism. The data were also analyzed using the Combenefit software (Di Veroli et al., 2016) which simultaneously assesses synergy/antagonism based on the assumption of non-interaction using three published models [Highest single agent (HSA), Bliss, and Loewe]. Dose-response curves were also included for each drug to generate a dose-response surface for the reference models, from which the experimental surface and modeled surface were then compared. At each combination, deviations in the experimental surface from the modeled surface were attributed to a percentage score indicating the degree of either synergy (increased effect) or antagonism (decreased effect). The “Contour” and “surface” plots were selected as graphical outputs for the synergy distribution.

### Cytotoxicity of Drug Combinations (WST-1 Assay)

The cytotoxic effect of the drug combinations was monitored by the colorimetric WST-1 assay according to the manufacturer’s instructions (Roche diagnostic, Mannheim, Germany). Briefly, A549 cells were seeded in 96-well plates and incubated for 24 h at 37°C. Thereafter, the cells were treated with ATR-002 and BXA combinations at the indicated concentrations. The cells were further incubated for 24 h followed by the addition of WST-1 reagent. The quantification of the formazan dye produced by metabolically active cells was measured using a scanning multi-well spectrophotometer at 450 and 650 nm for the reference wavelength after 4 h incubation.

### Statistical Analysis

Statistical analysis was performed using GraphPad Prism ver. 6 software. Data from three independent experiments are presented as the mean ± standard error (SEM). Dose-response as well as non-linear regression analysis for EC_50_ were calculated. Statistical differences were determined using unpaired *t*-test with Welch’s correction. *P*-values of ≤0.05 were considered statistically significant.

## Results

### Antiviral Activity of ATR-002 Against Different IAV Strains and Subtypes

**FIGURE 1:**
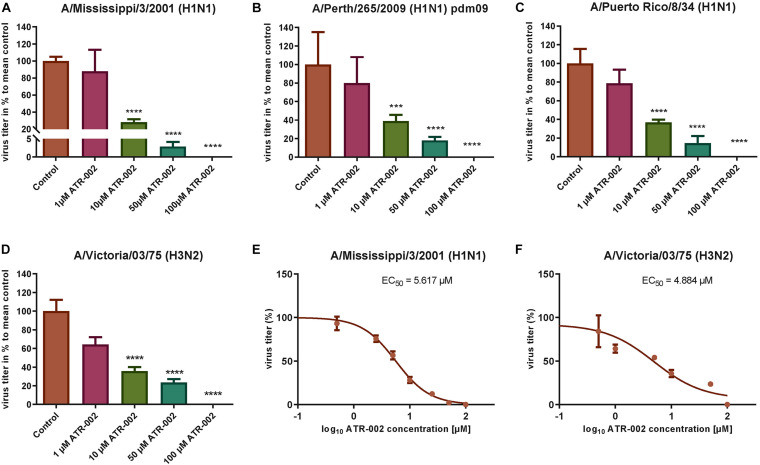
Antiviral potential of ATR-002 against different IAV strains and subtypes. **(A–D)** Dose-dependent inhibition was compared to control. Data are presented as mean ± SEM analyzed using unpaired *t*-test with Welch’s correction. Asterisks above columns represent the statistical significance (*p* < 0.05); **(E,F)** non-linear regression analysis in order to evaluate the EC_50_ values. Data are shown in log_10_ concentrations. All statistical tests were performed using GraphPad Prism 6.0 software (La Jolla, CA, United States).

To explore the antiviral potential of ATR-002 we determined virus titer reduction of different IAV strains and subtypes in the presence of defined ATR-002 concentrations (1, 10, 50, 100 μM), which are below the cytotoxic concentration 50% (CC_50_) for ATR-002 on A549 cells (271.8 μM) (Laure et al., 2020). At 100 μM ATR-002, we could not detect progeny virions of all investigated IAV strains (Figures 1A–D) and 50 μM ATR-002 still significantly (*P* > 0.0001) reduced the titer of the three H1N1-type IAV strains tested by 97.11 ± 0.72% for A/Mississippi/3/2001 (H1N1), 82 ± 1.49% for A/Perth/265/2009 (H1N1pdm09), and 85.5 ± 3.04% for A/Puerto Rico/8/34 (H1N1) (Figures 1A–C). A quite similar titer reduction by 76.4 ± 2.04% was observed for the H3N2-type A/Victoria/03/75 (H3N2) (Figure 1D). At 10 μM ATR-002 is also still able to reduce the titer by 71.81 ± 3.49, 61.14 ± 6.7, 63.21 ± 2.96% for the tested H1N1 strains respectively and by 64.29 ± 4.24% for an H3N2-type IAV. In contrast, 1 μM ATR-002 was insufficient to affect the titers of all examined strains significantly. Collectively, we demonstrate that ATR-002 has the capacity to block IAV replication across different strains and subtypes.

Next, the effective concentration 50% (EC_50_) for ATR-002 at which IAV progeny is reduced by 50%, was determined for two different IAV subtypes to be 5.62 μM for A/Mississippi/3/2001 (H1N1) and 4.884 μM for A/Victoria/03/75 (H3N2) (Figures 1E,F). These EC_50_ values are in line with our previous *in vitro* results for ATR-002 against an H1N1 (6.36 μM) and an H3N2 (4.84 μM) IAV strain (Laure et al., 2020). Equally, the corresponding selectivity index (SI = CC_50_/EC_50_) of 281 and 323 for A/Mississippi/3/2001 (H1N1) and A/Victoria/03/75 (H3N2), respectively, is comparable to the previously determined SI for a H1N1 subtype (SI: 248) and a H3N2 subtype (SI: 326) (Laure et al., 2020). These results indicate that ATR-002 is capable of impairing the replication of different IAV subtypes and strains with comparable efficiency.

### Growth Kinetics of Recombinant Viruses

**FIGURE 2:**
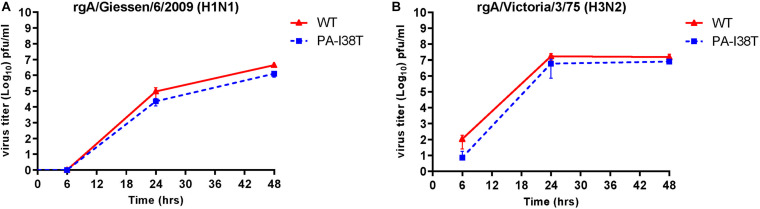
Replication curves of wild type viruses compared to the variant viruses with the indicated amino acid substitution in the PA protein. MDCK cells were infected (MOI = 0.001) with either wild type or PA-I38T mutant influenza virus **(A)** rgA/Giessen/6/2009 (H1N1) or **(B)** rgA/Victoria/3/75 (H3N2). The virus titers were determined in the supernatants at the respective time points. The data represent the mean ± SD values of three independent experiments. The statistical significance was evaluated using paired Student’s *t*-test (*p* < 0.05) and it was not significant.

In order to determine the replication kinetics of the recombinant viruses (rgH1N1-PA-I38T, rgH3N2-PA-I38T) carrying the PA-I38T mutation in comparison to their respective recombinant wild type (rgH1N1-WT, rgH3N2-WT), we analyzed the increase in virus titer in the supernatant of cells infected with the different viruses at 6, 24, and 48 hpi. For both pairs of recombinant viruses (PA-mutant, WT) we found similar replication dynamics even though the titer for the PA mutants (rgH1N1-/rgH3N2-PA-I38T) was always below the titer of the WT viruses by 0.28 – 0.62 log_10_ (Figures 2A,B). This is in agreement with published data indicating that the fitness of viruses isolated from BXA-treated persons, carrying the PA-I38T mutation, is reduced to a similar extent (Omoto et al., 2018). Taken together, the kinetics and the efficiencies in replication of the rgWT and the rgPA-mutants generated by reverse genetics reflect the data obtained from naturally occurring virus variants. Therefore, the recombinant viruses should possess the biological properties needed for further analysis.

### Antiviral Activity of ATR-002 Against BXA-Susceptible and Resistant IAV

**FIGURE 3:**
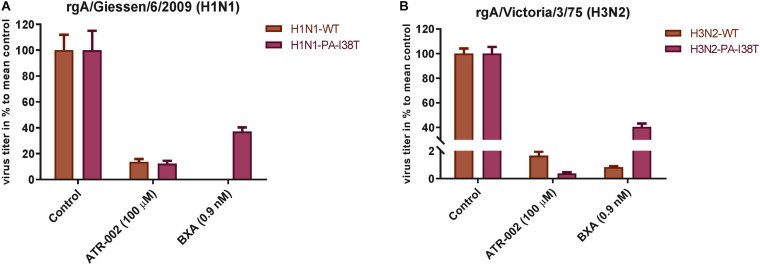
Antiviral activity of BXA or ATR-002 against both recombinant wild type and BXA-resistant influenza virus strains. **(A)** rgA/Giessen/06/2009 (H1N1), **(B)** rgA/Victoria/3/75 (H3N2). Confluent monolayer of MDCK-II cells were infected with the respective virus strains (MOI = 0.001) and incubated for 1 h. Subsequently, cells were treated by adding infection media containing either BXA or ATR-002 at the indicated concentrations. After 24 h, the virus titers were determined by focus assay. The data represent the mean ± SD values (*n* = 6) and analyzed using GraphPad Prism 6.0 software (La Jolla, CA, United States).

To confirm (i) reduced susceptibility toward BXA of the two recombinant PA-mutants (rgH1N1-PA-I38T, rgH3N2-PA-I38T) compared to the respective recombinant wild type viruses (rgH1N1-WT, rgH3N2-WT) and (ii) to further validate the antiviral activity of ATR-002 against the recombinant PA-mutants and WT viruses, we investigated the effect of ATR-002 versus BXA on the virus titer of the recombinant WT- and PA-mutant viruses 24 hpi compared to the untreated controls set to 100% (Figures 3A,B). The results demonstrate that BXA (0.9 nM) very efficiently inhibits rgH1N1-WT and rgH3N2-WT by 99.95 ± 0.02% and 99.2 ± 0.08%, respectively, while ATR-002 (100 μM) reduced the titers by 86.4 ± 2.24% and 98.4 ± 0.30%, respectively. In contrast, BXA reduced the titer of the PA-mutants rgH1N1-PA-I38T and rgH3N2-PA-I38T only by 63 ± 3.23% and 60 ± 2.70%, whereas ATR-002 still achieved a reduction by 87.80 ± 2.20% and 99.70 ± 0.10%, respectively. This corroborates that the PA-I38T mutation confers resistance toward BXA and clearly indicates that BXA-resistant viruses are still highly sensitive toward MEK inhibition by ATR-002.

### Synergy Results at Lower ATR-002 and BXA Dose Combinations

**FIGURE 4:**
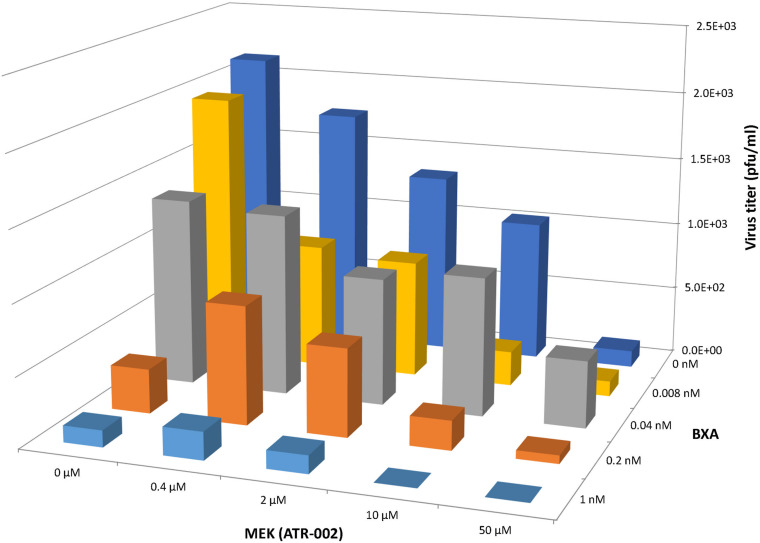
Antiviral effect of BXA/ATR-002 combinations. A549 cells were infected with A/Puerto Rico/8/34 (H1N1) (MOI = 0.001) and subsequently treated with the two inhibitors alone or in combination at the indicated concentrations. After 24 h, the virus titers in the supernatant from the infected cells were determined by focus assay. The plotted viral titers represent the mean values (*n* = 3) and indicate the increased or reduced antiviral activity.

Based on the above mentioned results we next investigated the synergistic potential of the combined treatment with BXA and ATR-002, as this would not only allow to impair BXA-resistant strains, but also to profit from possibly augmented virus inhibition due to the different modes of action simultaneously exerted by the two compounds. Hence, we designed a 4 × 4 matrix for the single/combined use of both compounds applying concentrations related to the determined EC_50_ values, against the model IAV strain A/Puerto Rico/8/34. Compared to the individual treatment with each drug virus titers indicated enhanced antiviral activity upon combining ATR-002 (0.4, 2, and 10 μM) with BXA (0.008 and 1.0 nM) (Figure 4). Moreover, there was no cytotoxicity associated with any of the drug combinations (no reduction in cell numbers by a companion assay of cells alone exposed to the same drug combinations) (Supplementary Figure 1).

To assess whether this observation was due to synergistic or additive effects of the two compounds, “CompuSyn” software was applied, which uses the “Combination Index” model (CI) that defines synergism or antagonism by referring to a quantitative degree of drug interaction to analyze the above mentioned data.

**FIGURE 5:**
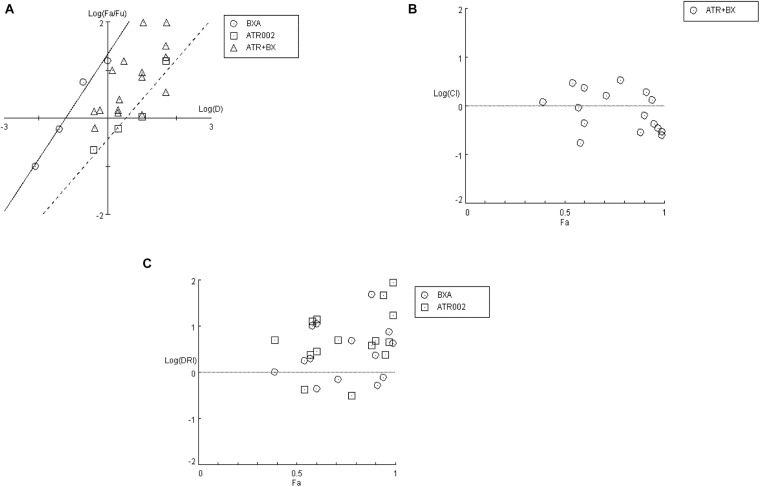
Analysis of BXA/ATR-002 combinations (Supplementary Table 4) with CompuSyn software. **(A)** Median-effect plot (ME-plot) for BXA and ATR-002. This plot generated by plotting the log values of *x* = D (dose) versus *y* = f_*a*_/f_*u*_ (fraction affected/fraction unaffected) and the *D*_*m*_ calculated according to the median-effect plot equation. **(B)** Logarithmic Combination Index Plot. Synergism/antagonism were plotted as the Log (CI) on the *y*-axis versus the Fraction affected (*F*_*a*_) on the *x*-axis. Combinations with CI value CI = 1 are additive and plotted on, or near the horizontal line, combinations with CI < 1 are synergistic and plotted below the horizontal line, and combinations with CI > 1 indicate antagonism and plotted above the horizontal line; **(C)** Fa-DRI plot of BXA and ATR-002 against influenza virus. DRI values above 1 [log (DRI) = 0] represents favorable drug dose reduction for each drug, DRI = 1 and <1 indicate neutral, and negative dose-reduction, respectively (Supplementary Table 5).

Firstly, a “Median-Effect” plot (ME-plot) was generated for both drugs (Figure 5A) to determine the quality of the obtained data by plotting the log values of the dose (D) versus the fraction affected/fraction unaffected (f_*a*_/f_*u*_) and the “Median Effect Dose” (*D*_*m*_) was calculated according to the median-effect plot equation “log (f_*a*_/f_*u*_) = m log (D) – m log (*D*_*m*_).” Primarily, as a step toward evaluating the conformity and the fidelity of the experimental data, the linear correlation coefficient (*r*) of the ME-plot was calculated for both drugs BXA (*r* = 0.99) and ATR-002 (*r* = 0.95), which reflects the validity of the obtained combination data. Moreover, *D*_*m*_, which under the described conditions determines the “Inhibitory Concentration 50” (IC_50_) value, was determined from the x-intercept of the ME-plot. The *D*_*m*_ values were found to be 0.0613 nM and 3.4884 μM for BXA and ATR-002, respectively.

Next, by applying a logarithmic “Combination Index” (CI) plot synergistic effects were determined (Figure 5B). As shown, there are 8 data points for specific drug combinations out of 16 that fall below the additive line (<1), revealing a synergistic effect with CI values ranging between 0.17 and 0.63 (Supplementary Table 4). Otherwise, data points of two combinations are near the additive line with a CI score of 0.91 – 1.18, indicating an additive effect and 6 further combinations are well above the additive line with CI scores of 1.31–3.32. These data indicate that the combination of ATR-002 and BXA results in a strong synergistic effect reflected by low CI values compared to the single-use concentration. Finally, using “CompuSyn” software, the potentially beneficial effect of a combined treatment was further analyzed by calculating the drug synergism as the “Dose-Reduction Index” (DRI) from a “Fa-DRI” plot (Chou-Martin plot) (Figure 5C). This method delineates how many folds the dose of each drug can be reduced when used in combination. DRI values above 1 describe a favorable “Dose Reduction” (DR). Explicitly, in order to achieve virus titer reduction by 57%, either 0.079 nM of BXA or 4.89 μM of ATR002 are required in a single-use (Supplementary Table 5). However, in combination about 1.98 fold less BXA and 2.44 fold less ATR-002 is required to achieve the same inhibition, which corresponds to 0.039 nM BXA and 2.004 μM ATR-002. Nevertheless, it should be noted that despite the advantage to overcome the toxic effects of a single drug application, DRI prediction can overestimate the doses predicted, as indicated by the shift from their empirical estimation. As such DRI would predict that either 4.23 nM BXA or 879.68 μM ATR-002 are needed in a single-use to achieve 99% virus titer reduction, but this prediction is not in line with our experimental data since this level of virus reduction could already be achieved with 1 nM BXA or 100 μM ATR-002 (Supplementary Table 5).

**FIGURE 6:**
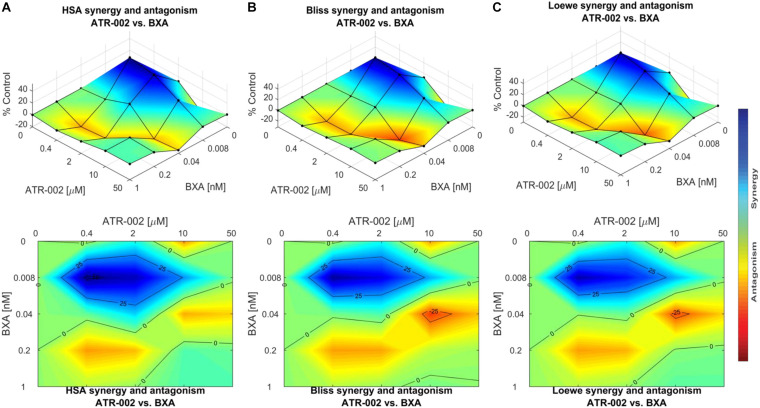
Data obtained from combinations of BXA/ATR-002 were tested in a 4 × 4 matrix and all values normalized to Mock-infected control (DMSO). Contour and surface plots were generated by Combenefit software upon processing data using three different synergy models: **(A)** HSA; **(B)** Bliss; **(C)** Loewe. The level of synergism or antagonism is represented with a color scale bar. Synergy is denoted by dark blue surface area and antagonism effect indicated by red surface area.

These data were confirmed using “Combenefit” software, allowing to compare the results of three different mathematical models (HSA, Bliss, and Loewe). These models evaluate the combinatory effect as scores, which reflect the distribution of synergism/antagonism and facilitate the comparison between the assigned models. Synergy levels were depicted as surface and contour plots, revealing that these models confirm the overall evaluation of the synergistic activity of both compounds against IAV at lower concentrations of each drug when compared to higher concentrations (Figure 6). Differences in the synergistic scores between the “HAS” model and the “Bliss” and “Loewe” model can be explained by the different hypotheses of each model and the corresponding equations. Taken together, compared to the individual doses of both compounds, lower drug dose combinations result in considerable synergy, as well as decreased additive effects in contrast to higher dose combinations.

## Discussion

Efforts to control IAV infection and the emergence of new strains are based on the development of vaccines and antiviral drugs. Of the currently worldwide available classes of antiviral drugs, the NAIs are used as the standard-of-care (SOC) treatment. Nevertheless, despite the low frequency of viruses with reduced susceptibility to NAI the necessity of early initiation of treatment together with the emergence of drug-resistant strains are considered as a major challenge for the use of these antivirals (Hayden and Shindo, 2019; Takashita et al., 2020). Furthermore, it was reported that the activity of oseltamivir carboxylate, the active metabolite of oseltamivir, is associated with delayed antiviral effects and clinical resolution during the treatment of Influenza B viruses (IBV) infections when compared to the treatment of IAV infections (Kawai et al., 2006; Sugaya et al., 2007; Hayden, 2009). Among different antiviral approaches, BXA is not only effective against human IAV and IBV but also avian and porcine subtypes and has been approved in Japan and United States (Hayden et al., 2018; Heo, 2018; Omoto et al., 2018; Mishin et al., 2019; Takashita et al., 2019). Despite the robustness of BXA as a single-dose antiviral drug, BXA treatment has resulted in a high level of viral resistance with 76- to 120-fold reduced BXA susceptibility in Japan (Takashita et al., 2019) and 4- to 10-fold in the United States (Gubareva et al., 2019). Moreover, another study reported that even though the IC_50_ of BXA against a wide type pandemic H1N1-type IAV (WT/H1N1 pdm09) was 0.42 ± 0.37 nM, its IC_50_ against the I38T variant was 41.96 ± 9.42 nM; and the IC_50_ value for WT H3N2 virus was 0.66 ± 0.17 nM, but 139.73 ± 24.97 nM for the I38t mutant (Checkmahomed et al., 2020). Altogether, the above mentioned limitations highlight the urgent need for alternative approaches.

In response to an influenza virus infection, several intracellular signal transduction pathways are triggered and play a key role in the viral replication cycle. Earlier *in vitro* studies reported the applicability of MEK-inhibitors as promising antiviral compounds showing that inhibiting MEK leads to nuclear retention of vRNPs, thereby suppressing viral replication (Pleschka et al., 2001). Furthermore, MEK inhibition exhibited a high antiviral potential against diverse IAV/IBV strains including H5N1-type highly pathogenic avian influenza viruses (HPAIV) (Ludwig et al., 2004; Droebner et al., 2011; Haasbach et al., 2017). Adverse effects are often considered the main concern upon inhibition of cellular signaling in the treatment of seasonal influenza. As suggested earlier, these concerns could be minimized by either the short-term treatment of IV-infected patients compared to the long-term and repeated treatment of cancer patients or by reducing the dose while maintaining a strong antiviral activity through the combination of the signaling inhibitor with other direct-acting antiviral compounds (Planz, 2013). Along this line, we extended our investigations of the antiviral activity of ATR-002.

Herein, we report that ATR-002 (50 μM) is able to diminish IAV replication reducing the viral titer by 82 ± 1.49% to 97.8% for different H1N1 subtypes and by 76.4 ± 2.04% for a H3N2 subtype (Figure 1) with similar EC_50_ values from 5.617 μM to 4.884 μM for H1N1- and H3N2-type IAV strains, respectively. These results demonstrate that ATR-002 efficiently blocks IAV replication across different strains and subtypes and corroborates our previously published data (Laure et al., 2020) demonstrating the *in vitro* and *in vivo* efficacy of ATR-002 or CI-1040, against IAV.

To evaluate whether the indirectly antiviral acting host-targeting MEK inhibitor could overcome viral resistance of IAV carrying substitutions mounted against the directly acting antiviral BXA, the inhibitory activity of ATR-002 was validated against two recombinant IAVs, a H1N1- and a H3N2-strain, both either wild type or carrying the BXA resistance-associated PA-I38T mutation. In regard to comparability and to exclude unwanted additional mutations/differences, we generated these viruses as recombinant wild type and mutant variants (rgH1N1-WT/- PA-I38T, rgH3N2-WT/- PA-I38T) allowing us to perform unbiased comparison of the activities exerted by BXA or ATR-002. Notably, both mutant strains replicate with a lower efficiency compared to their wild type counterpart, as described for isolates obtained from BXA-treated individuals (Omoto et al., 2018) (Figure 2), indicating that the recombinant viruses reflect the biological properties of the natural isolates. On the contrary, in a growth-competition experiment, the growth-kinetics analysis revealed that the I38T substitution impairs the replication fitness of A/H1N1pdm viruses, while this mutation does not alter the fitness of A/H3N2 viruses *in vitro*. Moreover, the A/H1N1pdm viral replication fitness of the original clinical isolates may be restored by a concomitant compensatory mutation(s) (Imai et al., 2020). The same study also showed no substantial change in the replication fitness between the mutant and wild type viruses of both IAV subtypes *in vivo*.

The PA-I38T mutation was described to be associated with reduced IAV susceptibility to BXA (Omoto et al., 2018; Gubareva et al., 2019) and IAV variants harboring an I38T, I38F, or I38M substitution showed reduced BXA susceptibility during phase 2 and phase 3 trials, respectively (Hayden et al., 2018). Furthermore, IAV circulating during the 2016/2017 and 2017/2018 seasons in the United States gained amino acid substitutions in the PA-I38 position conferring 4–10-fold reduction in BXA susceptibility (Gubareva et al., 2019). Here we demonstrate that, despite the potent effect of BXA against both WT strains, its antiviral effect was significantly reduced by ∼40% against the mutant strains, while ATR-002 still impaired the PA-I38T mutants to a similar extent as the respective wild type strains (Figure 3). Based on our previous findings, these results underline that targeting cellular MEK can overcome viral resistance mounted against directly antiviral acting compounds.

Notwithstanding the single use of antiviral compounds, combination therapy may be useful, not only to increase the antiviral potential of the therapy but also to counteract the limitations for SOC mono-therapy, such as rapid viral resistance against direct-acting antiviral compounds and may enhance the clinical benefits by minimizing the dose-related toxicity (Govorkova and Webster, 2010). Additionally, with regard to more severe scenarios, e.g., severe infection or immunocompromised individuals, the combination treatment presumably provides meaningful benefits beyond those attained with current agents in many other situations like the delayed treatment with SOC antivirals following symptom onset that the SOC inefficient to reduce severity, illness duration, or duration of virus detection. We addressed a combination therapy approach by simultaneously employing two different antiviral mechanisms, e.g., inhibition of nuclear vRNP export and the PA endonuclease activity. Compared to mono-therapy, this combination regimen reduced the IAV titer more efficiently. Importantly, the combined BXA/ATR-002 treatment achieved a synergistic effect with CI values ranging from 0.17 to 0.63, especially when both compounds were combined in concentrations below their single-use concentration. The most commonly used approaches to estimate synergism can be divided into effect-based approaches and dose-effect-based approaches. Each approach has practical advantages and limitations. Yet, in the absence of a reference methodology suitable for all biomedical situations, the analysis of drug combinations should benefit from a collective, appropriate, and rigorous application of the concepts and methods (Foucquier and Guedj, 2015). Along this line, we have chosen the most commonly used methods as representative analysis models and synergy was then further validated by three different models, which confirmed that our estimation of synergism is in line with the interpretation of the CI model.

Previously, another example for a combinatorial drug treatment approach based on cell-culture assays showed that nitazoxanide (blocking viral hemagglutinin maturation) exerts a synergistic effect when combined with oseltamivir or zanamivir (targeting the viral neuraminidase) (Belardo et al., 2015). Also, in a triple combination antiviral regime, the activity of oseltamivir, amantadine (targeting the viral M2 protein), and ribavirin (targeting the RdRp) was evaluated against oseltamivir- and amantadine-resistant seasonal IAV strains and exhibited a higher synergistic effect than double combinations (Nguyen et al., 2010). In support of such studies that employ combinations of divergent antiviral mechanisms, we have previously investigated the effect of oseltamivir combined with different MEK-inhibitors and could additionally demonstrate a synergistic effect (Haasbach et al., 2013).

## Conclusion

In regard to our earlier studies, it is tempting to speculate that repurposing the MEK- inhibitor ATR-002 may represent an attractive approach to counteract the limitations of currently approved drugs against influenza viruses, that all target a viral function, in order to successfully address viral resistance. Moreover, these results substantiate both scenarios for the use of ATR-002 (i) in a mono-therapy, as well as (ii) in a combination approach (together with SOC drugs) targeting different viral and host factors. To address the safety of cell signaling inhibitors as antivirals, it should be noted that ATR-002 was successfully tested in phase I clinical trial demonstrating no considerable adverse effects, and is now in progress for further development.

## Data Availability Statement

The original contributions presented in the study are included in the article/Supplementary Material. Further inquiries can be directed to the corresponding author/s.

## Author Contributions

HH, MMS, AM, SP, and OP participated in the conceptualization and design of the study. HH and MMS performed experimental drug testing experiments. HH performed drug combination treatment experiments. MMS and AM conducted the recombinant virus experiments. HH, MMS, AM, SP, and OP analyzed and interpreted the data. HH drafted the article. AM, SP, and OP administered the project and supervised the study. All authors revised and approved the submitted manuscript.

## Conflict of Interest

SP and OP are members of the German FluResearchNet, a nationwide research network on zoonotic influenza and are consultants for Atriva Therapeutics GmbH. The remaining authors declare that the research was conducted in the absence of any commercial or financial relationships that could be construed as a potential conflict of interest.

## Supplementary Material

Supplementary Figure 1 Evaluation of the cytotoxic effect of BXA and ATR-002 combinations.

Supplementary Table 1 Primers used to generate the PA-I38T mutation via site direct mutagenesis.

Supplementary Table 2 Sequencing primers used for complete sequencing of pMP-PA plasmids.

Supplementary Table 3 Primers used in RT-PCR and sequencing.

Supplementary Table 4 Combination Index (CI) values for drug combos.

Supplementary Table 5 Drug dose reduction (DRI) data example of BXM and ATR-002 prediction combo.

## References

[B1] Belardo G. Cenciarelli O. La Frazia S. Rossignol J. F. Santoro M. G. (2015). Synergistic effect of nitazoxanide with neuraminidase inhibitors against influenza A viruses in vitro. *Antimicrob. Agents Chemother.* 59 1061–1069. 10.1128/AAC.03947-14 25451059 PMC4335909

[B2] Checkmahomed L. M’Hamdi Z. Carbonneau J. Venable M. C. Baz M. Abed Y. (2020). Impact of the Baloxavir-Resistant Polymerase Acid I38T Substitution on the Fitness of Contemporary Influenza A(H1N1)pdm09 and A(H3N2) Strains. *J. Infect. Dis.* 221 63–70. 10.1093/infdis/jiz418 31419295 PMC6910874

[B3] Chou T. Martin N. (2005). *CompuSyn for drug combinations: PC software and user’s guide: a computer program for quantitation of synergism and antagonism in drug combinations, and the determination of IC50 and ED50 and LD50 values.* Paramus, NJ: ComboSyn.

[B4] Chou T. C. (2010). Drug combination studies and their synergy quantification using the Chou-Talalay method. *Cancer Res.* 70 440–446. 10.1158/0008-5472.can-09-1947 20068163

[B5] Chou T. C. Talalay P. (1984). Quantitative analysis of dose-effect relationships: the combined effects of multiple drugs or enzyme inhibitors. *Adv. Enzyme Regul.* 22 27–55. 10.1016/0065-2571(84)90007-4 6382953

[B6] Clark M. P. Ledeboer M. W. Davies I. Byrn R. A. Jones S. M. Perola E. (2014). Discovery of a novel, first-in-class, orally bioavailable azaindole inhibitor (VX-787) of influenza PB2. *J. Med. Chem.* 57 6668–6678. 10.1021/jm5007275 25019388

[B7] Di Veroli G. Y. Fornari C. Wang D. Mollard S. Bramhall J. L. Richards F. M. (2016). Combenefit: an interactive platform for the analysis and visualization of drug combinations. *Bioinformatics* 32 2866–2868. 10.1093/bioinformatics/btw230 27153664 PMC5018366

[B8] Droebner K. Pleschka S. Ludwig S. Planz O. (2011). Antiviral activity of the MEK-inhibitor U0126 against pandemic H1N1v and highly pathogenic avian influenza virus in vitro and in vivo. *Antiviral. Res.* 92 195–203. 10.1016/j.antiviral.2011.08.002 21854809

[B9] Duwe S. (2017). Influenza viruses - antiviral therapy and resistance. *GMS Infect. Dis.* 5:Doc04. 10.3205/id000030 30671326 PMC6301739

[B10] Fodor E. (2013). The RNA polymerase of influenza a virus: mechanisms of viral transcription and replication. *Acta Virol.* 57 113–122. 10.4149/av_2013_02_113 23600869

[B11] Foucquier J. Guedj M. (2015). Analysis of drug combinations: current methodological landscape. *Pharmacol. Res. Perspect.* 3:e00149. 10.1002/prp2.149 26171228 PMC4492765

[B12] Fremin C. Meloche S. (2010). From basic research to clinical development of MEK1/2 inhibitors for cancer therapy. *J. Hematol. Oncol.* 3:8. 10.1186/1756-8722-3-8 20149254 PMC2830959

[B13] Govorkova E. A. Webster R. G. (2010). Combination chemotherapy for influenza. *Viruses* 2 1510–1529. 10.3390/v2081510 21994692 PMC3185732

[B14] Gubareva L. V. Mishin V. P. Patel M. C. Chesnokov A. Nguyen H. T. De La Cruz J. (2019). Assessing baloxavir susceptibility of influenza viruses circulating in the United States during the 2016/17 and 2017/18 seasons. *Euro. Surveill.* 24:1800666. 10.2807/1560-7917.ES.2019.24.3.1800666 30670144 PMC6344838

[B15] Haasbach E. Hartmayer C. Planz O. (2013). Combination of MEK inhibitors and oseltamivir leads to synergistic antiviral effects after influenza A virus infection in vitro. *Antiviral. Res.* 98 319–324. 10.1016/j.antiviral.2013.03.006 23523553

[B16] Haasbach E. Muller C. Ehrhardt C. Schreiber A. Pleschka S. Ludwig S. (2017). The MEK-inhibitor CI-1040 displays a broad anti-influenza virus activity in vitro and provides a prolonged treatment window compared to standard of care in vivo. *Antiviral. Res.* 142 178–184. 10.1016/j.antiviral.2017.03.024 28377100

[B17] Hayden F. (2009). Developing new antiviral agents for influenza treatment: what does the future hold? *Clin. Infect. Dis.* 48 S3–S13. 10.1086/591851 19067613

[B18] Hayden F. G. Shindo N. (2019). Influenza virus polymerase inhibitors in clinical development. *Curr. Opin. Infect. Dis.* 32 176–186. 10.1097/QCO.0000000000000532 30724789 PMC6416007

[B19] Hayden F. G. Sugaya N. Hirotsu N. Lee N. de Jong M. D. Hurt A. C. (2018). Baloxavir Marboxil for Uncomplicated Influenza in Adults and Adolescents. *N. Engl. J. Med.* 379 913–923. 10.1056/NEJMoa1716197 30184455

[B20] Heo Y. A. (2018). Baloxavir: First Global Approval. *Drugs* 78 693–697. 10.1007/s40265-018-0899-1 29623652

[B21] Hirotsu N. Sakaguchi H. Sato C. Ishibashi T. Baba K. Omoto S. (2019). Baloxavir marboxil in Japanese pediatric patients with influenza: safety and clinical and virologic outcomes. *Clin. Infect. Dis*. 71 971–981. 10.1093/cid/ciz908 31538644 PMC7428393

[B22] Imai M. Yamashita M. Sakai-Tagawa Y. Iwatsuki-Horimoto K. Kiso M. Murakami J. (2020). Influenza A variants with reduced susceptibility to baloxavir isolated from Japanese patients are fit and transmit through respiratory droplets. *Nat. Microbiol.* 5 27–33. 10.1038/s41564-019-0609-0 31768027 PMC13014278

[B23] Jones J. C. Kumar G. Barman S. Najera I. White S. W. Webby R. J. (2018). Identification of the I38T PA Substitution as a Resistance Marker for Next-Generation Influenza Virus Endonuclease Inhibitors. *MBio* 9 430–418e. 10.1128/mBio.00430-18 29691337 PMC5915737

[B24] Kawai N. Ikematsu H. Iwaki N. Maeda T. Satoh I. Hirotsu N. (2006). A comparison of the effectiveness of oseltamivir for the treatment of influenza A and influenza B: a Japanese multicenter study of the 2003-2004 and 2004-2005 influenza seasons. *Clin. Infect. Dis.* 43 439–444. 10.1086/505868 16838232

[B25] Kim W. Y. Young Suh G. Huh J. W. Kim S. H. Kim M. J. Kim Y. S. (2011). Triple-combination antiviral drug for pandemic H1N1 influenza virus infection in critically ill patients on mechanical ventilation. *Antimicrob. Agents Chemother.* 55 5703–5709. 10.1128/AAC.05529-11 21968371 PMC3232815

[B26] Laure M. Hamza H. Koch-Heier J. Quernheim M. Muller C. Schreiber A. (2020). Antiviral efficacy against influenza virus and pharmacokinetic analysis of a novel MEK-inhibitor, ATR-002, in cell culture and in the mouse model. *Antiviral. Res.* 178:104806. 10.1016/j.antiviral.2020.104806 32304723

[B27] Lauring A. S. Andino R. (2010). Quasispecies theory and the behavior of RNA viruses. *PLoS Pathog.* 6:e1001005. 10.1371/journal.ppat.1001005 20661479 PMC2908548

[B28] Lee S. M. Yen H. L. (2012). Targeting the host or the virus: current and novel concepts for antiviral approaches against influenza virus infection. *Antiviral. Res.* 96 391–404. 10.1016/j.antiviral.2012.09.013 23022351 PMC7132421

[B29] Lorusso P. M. Adjei A. A. Varterasian M. Gadgeel S. Reid J. Mitchell D. Y. (2005). Phase I and pharmacodynamic study of the oral MEK inhibitor CI-1040 in patients with advanced malignancies. *J. Clin. Oncol.* 23 5281–5293. 10.1200/JCO.2005.14.415 16009947

[B30] Ludwig S. (2011). Disruption of virus-host cell interactions and cell signaling pathways as an anti-viral approach against influenza virus infections. *Biol. Chem.* 392 837–847. 10.1515/BC.2011.121 21823902

[B31] Ludwig S. Wolff T. Ehrhardt C. Wurzer W. J. Reinhardt J. Planz O. (2004). MEK inhibition impairs influenza B virus propagation without emergence of resistant variants. *FEBS Lett.* 561 37–43. 10.1016/S0014-5793(04)00108-5 15013748

[B32] Ma W. Brenner D. Wang Z. Dauber B. Ehrhardt C. Högner K. (2010). The NS Segment of an H5N1 Highly Pathogenic Avian Influenza Virus (HPAIV) Is Sufficient To Alter Replication Efficiency, Cell Tropism, and Host Range of an H7N1 HPAIV. *J. Virol.* 84:2122. 10.1128/JVI.01668-09 20007264 PMC2812369

[B33] Marjuki H. Alam M. I. Ehrhardt C. Wagner R. Planz O. Klenk H. D. (2006). Membrane accumulation of influenza A virus hemagglutinin triggers nuclear export of the viral genome via protein kinase Calpha-mediated activation of ERK signaling. *J. Biol. Chem.* 281 16707–16715. 10.1074/jbc.M510233200 16608852

[B34] Marjuki H. Yen H. L. Franks J. Webster R. G. Pleschka S. Hoffmann E. (2007). Higher polymerase activity of a human influenza virus enhances activation of the hemagglutinin-induced Raf/MEK/ERK signal cascade. *Virol. J.* 4:134. 10.1186/1743-422X-4-134 18053252 PMC2222635

[B35] Matrosovich M. Matrosovich T. Garten W. Klenk H.-D. (2006). New low-viscosity overlay medium for viral plaque assays. *Virol. J.* 3:63. 10.1186/1743-422X-3-63 16945126 PMC1564390

[B36] Mishin V. P. Patel M. C. Chesnokov A. De La Cruz J. Nguyen H. T. Lollis L. (2019). Susceptibility of Influenza A, B, C, and D Viruses to Baloxavir. *Emerg. Infect. Dis.* 25 1969–1972. 10.3201/eid2510.190607 31287050 PMC6759234

[B37] Mostafa A. Kanrai P. Ziebuhr J. Pleschka S. (2013). Improved dual promotor-driven reverse genetics system for influenza viruses. *J. Virol. Methods* 193 603–610. 10.1016/j.jviromet.2013.07.021 23886561

[B38] Muhlbauer D. Dzieciolowski J. Hardt M. Hocke A. Schierhorn K. L. Mostafa A. (2015). Influenza virus-induced caspase-dependent enlargement of nuclear pores promotes nuclear export of viral ribonucleoprotein complexes. *J. Virol.* 89 6009–6021. 10.1128/JVI.03531-14 25810542 PMC4442457

[B39] Nguyen J. T. Hoopes J. D. Le M. H. Smee D. F. Patick A. K. Faix D. J. (2010). Triple combination of amantadine, ribavirin, and oseltamivir is highly active and synergistic against drug resistant influenza virus strains in vitro. *PLoS One* 5:e9332. 10.1371/journal.pone.0009332 20179772 PMC2825274

[B40] Noshi T. Kitano M. Taniguchi K. Yamamoto A. Omoto S. Baba K. (2018). In vitro characterization of baloxavir acid, a first-in-class cap-dependent endonuclease inhibitor of the influenza virus polymerase PA subunit. *Antiviral. Res.* 160 109–117. 10.1016/j.antiviral.2018.10.008 30316915

[B41] Obayashi E. Yoshida H. Kawai F. Shibayama N. Kawaguchi A. Nagata K. (2008). The structural basis for an essential subunit interaction in influenza virus RNA polymerase. *Nature* 454 1127–1131. 10.1038/nature07225 18660801

[B42] Omoto S. Speranzini V. Hashimoto T. Noshi T. Yamaguchi H. Kawai M. (2018). Characterization of influenza virus variants induced by treatment with the endonuclease inhibitor baloxavir marboxil. *Sci. Rep.* 8:9633. 10.1038/s41598-018-27890-4 29941893 PMC6018108

[B43] Pflug A. Lukarska M. Resa-Infante P. Reich S. Cusack S. (2017). Structural insights into RNA synthesis by the influenza virus transcription-replication machine. *Virus Res.* 234 103–117. 10.1016/j.virusres.2017.01.013 28115197

[B44] Pinto R. Herold S. Cakarova L. Hoegner K. Lohmeyer J. Planz O. (2011). Inhibition of influenza virus-induced NF-kappaB and Raf/MEK/ERK activation can reduce both virus titers and cytokine expression simultaneously in vitro and in vivo. *Antiviral. Res.* 92 45–56. 10.1016/j.antiviral.2011.05.009 21641936

[B45] Pires de Mello C. P. Drusano G. L. Adams J. R. Shudt M. Kulawy R. (2018). Oseltamivir-zanamivir combination therapy suppresses drug-resistant H1N1 influenza A viruses in the hollow fiber infection model (HFIM) system. *Eur. J. Pharm. Sci.* 111 443–449. 10.1016/j.ejps.2017.10.027 29079337 PMC5694373

[B46] Planz O. (2013). Development of cellular signaling pathway inhibitors as new antivirals against influenza. *Antiviral. Res.* 98 457–468. 10.1016/j.antiviral.2013.04.008 23603495

[B47] Pleschka S. (2013). Overview of influenza viruses. *Curr. Top Microbiol. Immunol.* 370 1–20. 10.1007/82_2012_272 23124938

[B48] Pleschka S. Wolff T. Ehrhardt C. Hobom G. Planz O. Rapp U. R. (2001). Influenza virus propagation is impaired by inhibition of the Raf/MEK/ERK signalling cascade. *Nat. Cell Biol.* 3 301–305. 10.1038/35060098 11231581

[B49] Schrader T. Dudek S. E. Schreiber A. Ehrhardt C. Planz O. Ludwig S. (2018). The clinically approved MEK inhibitor Trametinib efficiently blocks influenza A virus propagation and cytokine expression. *Antiviral. Res.* 157 80–92. 10.1016/j.antiviral.2018.07.006 29990517

[B50] Sebolt-Leopold J. S. (2004). MEK inhibitors: a therapeutic approach to targeting the Ras-MAP kinase pathway in tumors. *Curr. Pharm. Des.* 10 1907–1914. 10.2174/1381612043384439 15180527

[B51] Sebolt-Leopold J. S. Dudley D. T. Herrera R. Van Becelaere K. Wiland A. Gowan R. C. (1999). Blockade of the MAP kinase pathway suppresses growth of colon tumors in vivo. *Nat. Med.* 5 810–816. 10.1038/10533 10395327

[B52] Song M. S. Kumar G. Shadrick W. R. Zhou W. Jeevan T. Li Z. (2016). Identification and characterization of influenza variants resistant to a viral endonuclease inhibitor. *Proc. Natl. Acad. Sci. U. S. A.* 113 3669–3674. 10.1073/pnas.1519772113 26976575 PMC4822642

[B53] Sugaya N. Mitamura K. Yamazaki M. Tamura D. Ichikawa M. Kimura K. (2007). Lower clinical effectiveness of oseltamivir against influenza B contrasted with influenza A infection in children. *Clin. Infect. Dis.* 44 197–202. 10.1086/509925 17173216

[B54] Takashita E. Daniels R. S. Fujisaki S. Gregory V. Gubareva L. V. Huang W. (2020). Global update on the susceptibilities of human influenza viruses to neuraminidase inhibitors and the cap-dependent endonuclease inhibitor baloxavir, 2017–2018. *Antiviral. Res.* 175:104718. 10.1016/j.antiviral.2020.104718 32004620

[B55] Takashita E. Kawakami C. Morita H. Ogawa R. Fujisaki S. Shirakura M. (2019). Detection of influenza A(H3N2) viruses exhibiting reduced susceptibility to the novel cap-dependent endonuclease inhibitor baloxavir in Japan, December 2018. *Euro. Surveill.* 24:1800698. 10.2807/1560-7917.ES.2019.24.3.1800698 30670142 PMC6344841

[B56] Tecle H. Shao J. Li Y. Kothe M. Kazmirski S. Penzotti J. (2009). Beyond the MEK-pocket: can current MEK kinase inhibitors be utilized to synthesize novel type III NCKIs? Does the MEK-pocket exist in kinases other than MEK? *Bioorg. Med. Chem. Lett.* 19 226–229. 10.1016/j.bmcl.2008.10.108 19019675

[B57] Vanderlinden E. Vrancken B. Van Houdt J. Rajwanshi V. K. Gillemot S. Andrei G. (2016). Distinct Effects of T-705 (Favipiravir) and Ribavirin on Influenza Virus Replication and Viral RNA Synthesis. *Antimicrob. Agents Chemother.* 60 6679–6691. 10.1128/AAC.01156-16 27572398 PMC5075073

