# Supplementary material for: Improved in vitro Efficacy of Baloxavir Marboxil Against Influenza A Virus Infection by Combination Treatment With the MEK Inhibitor ATR-002
Source: Front Microbiol. 2021 Feb 12;12:611958. doi: 10.3389/fmicb.2021.611958 (PMC7928405; doi:10.3389/fmicb.2021.611958)
Supplement: Supplementary Figure 1 — Evaluation of the cytotoxic effect of BXA and ATR-002 combinations. [file Data_Sheet_1.PDF]

Supplementary figures

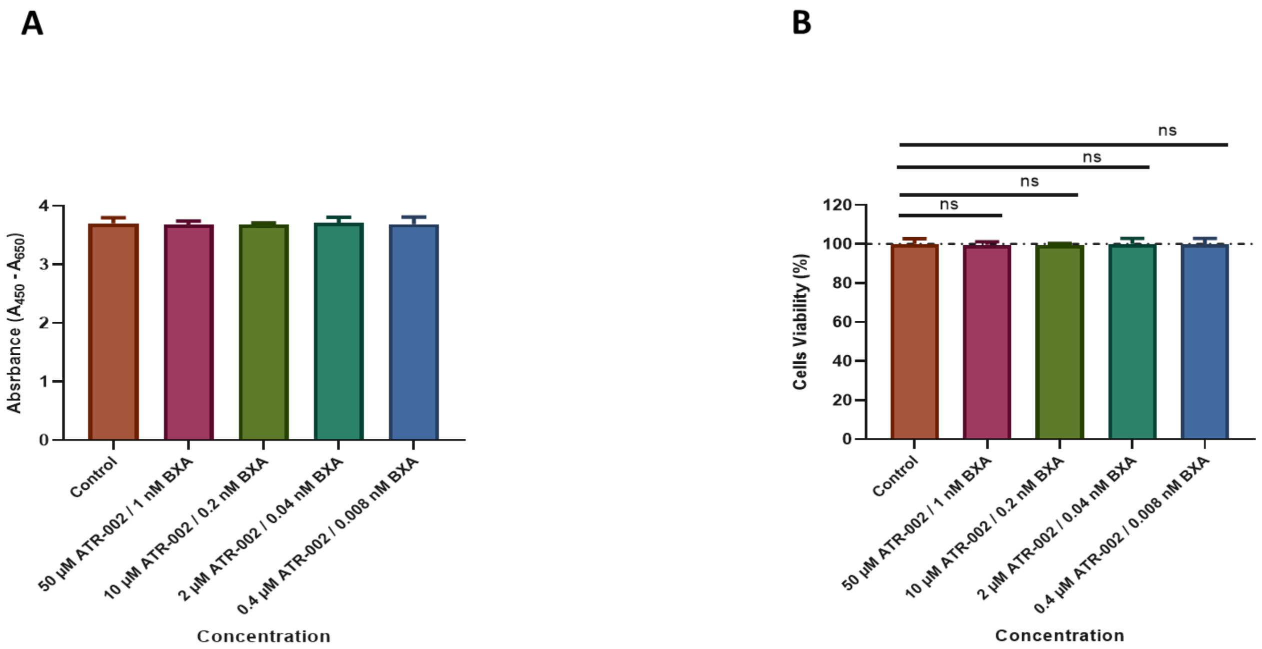

Fig. S1: Evaluation of the cytotoxic effect of BXA and ATR-002 combinations. The bar graphs depict the cytotoxicity data determined by the WST-1 assay. A549 cells were treated with the highest relevant combinations of both tested drugs (ATR-002/BXA) (50 µM / 1 nM; 10 µM / 0.2 nM; 2 µM/0.04 nM; 0.4 µM / 0.008 nM) and the viability was tested as indicated in the material and methods section. Values of the WST-1 assay are presented as (A) normalized absorbance values to the reference wavelength and (B) the viability percentage with control as 100 %. The data represent the mean ±SD (n=3) and the statistical significance was evaluated using unpaired t-test with Welch's correction compared to the control (ns: not significant). The data were analyzed using GraphPad Prism 6.0 software (La Jolla California, USA).
