# Supplementary material for: Improved in vitro Efficacy of Baloxavir Marboxil Against Influenza A Virus Infection by Combination Treatment With the MEK Inhibitor ATR-002
Source: Front Microbiol. 2021 Feb 12;12:611958. doi: 10.3389/fmicb.2021.611958 (PMC7928405; doi:10.3389/fmicb.2021.611958)
Supplement: Supplementary Table 1 — Primers used to generate the PA-I38T mutation via site direct mutagenesis. [file Data_Sheet_2.PDF]

Table (1): Primers used to generate the PA-I38T mutation via site direct mutagenesis

**Primer name**	**Sequence (5` to 3`)**
PA-H1-09-I38T-F	CTAACAAGTTTGCTGCAACATGCACACATTTGGAAG
PA-H1-09-I38T-R	CTTCCAAATGTGTGCATGTTGCAGCAAACTTGTTAG
PA-H3Vic-I38T-F	CAAACAAATTTGCAGCAACATGCACTCACTTGGAGG
PA-H3Vic-I38T-R	CCTCCAAGTGAGTGCATGTTGCTGCAAATTTGTTTG

Table (2): Sequencing primers used for complete sequencing of pMP-PA plasmids

Primer name	Sequence (5` to 3`)
SPA-1198R	GCTCTGGCTCATCACTGTCATACT
SPA-558F	TCTATGGGATTCCTTTCGTCAGTC
SPA-1982R	TCAGCCGAAAACCCCTCAAGT
SPA-1117F	AATATGGCACCAGAAAAAGTAGAC
PA-H3N2Vict-1892R	TCCCCAATGGGCCATGTTTC
PA-H3N2Vict-990F	CGTCAAACCACACGAAAGGG
PA-H3N2Vict-491F	CCACAAAGGCCGACTACACT
PA-H3N2Vict-1154R	TCTGGTGCCATGTTCTCACC
pHW2000R	GCTCCGTGTGTGGCTGCGAT
pHW2000F	GGTAAATGGCCCGCCTGGCA

Table (3): Primers used in RT-PCR and sequencing

**Primer name**	**Sequence (5` to 3`)**
Primers used for RT-PCR	
Bm-PA-1 F	TATTCGTCTCAGGGAGCGAAAGCAGGTAC
Bm-PA-2233R	ATATCGTCTCGTATTAGTAGAAACAAGGTACTT
Primers used for sequencing	
Bm-PA-1 F	TATTCGTCTCAGGGAGCGAAAGCAGGTAC
SPA-1198R	GCTCTGGCTCATCACTGTCATACT
PA-H3N2Vict-1154R	TCTGGTGCCATGTTCTCACC

Table (4): Combination Index (CI) values for drug combos

Dose BXM	Dose ATR-002	Effect*	CI
0.0080	0.4	0.58	0.17469
1.0	10.0	0.99	0.24757
0.0080	10.0	0.88	0.28142
1.0	50.0	0.99	0.29305
0.2	50.0	0.97	0.35104
0.0080	50.0	0.95	0.42303
0.0080	2.0	0.6	0.44177
0.2	10.0	0.9	0.63435
0.04	2.0	0.57	0.91172
0.04	0.4	0.39	1.18204
1.0	2.0	0.94	1.31281
0.2	2.0	0.71	1.62481
1.0	0.4	0.91	1.94132
0.2	0.4	0.6	2.31597
0.04	10.0	0.54	2.92652
0.04	50.0	0.78	3.32808

* % reduction in virus titer

Table (5): Drug dose reduction (DRI) data example of BXM and ATR-002 prediction combo

Fa*	Dose BXM (nM)	Dose ATR002 (µM)	DRI BXM	DRI ATR002
0.99	4.23358[a]	879.684[a]	4.23358	17.5937
0.97	1.50936	228.8[a]	7.54681	4.57599
0.95	0.92466	120.664[a]	115.583	2.41327
0.94	0.77405	95.6649	0.77405	47.8325
0.91	0.517	56.4779	0.517	141.195
0.9	0.4644	49.0947	2.32201	4.90947
0.88	0.38452	38.3704	48.0652	3.83704
0.78	0.1968	16.0009	4.91991	0.32002
0.71	0.1399	10.2473	0.69948	5.12367
0.6	0.08906	5.68262	11.133	2.84131
0.58	0.08253	5.14432	10.316	12.8608
0.57	0.07947	4.89711	1.98684	2.44855
0.54	0.07105	4.23089	1.77634	0.42309

*% reduction in virus titer

[a]Predicted dose that shift from its empirical estimation
